# Seasonal trends and population status of the highly threatened *Pteropus livingstonii* in the Comoros archipelago

**DOI:** 10.1186/s12862-024-02255-w

**Published:** 2024-05-19

**Authors:** Isabella Mandl, Amelaid Houmadi, Ishaka Said, Badrane Ben Ali Abdou, Nastazia Mohamed, Abdoulkader Fardane, Samirou Soulaïmana, Misbahou Mohamed, Ben Anthoy M., Hugh Doulton

**Affiliations:** 1 Department of Botany and Biodiversity Research, University of Vienna, Vienna, Austria; 2 NGO Dahari, Hombo - Mutsamudu, Anjouan Comoros; 3 Parc National de Mohéli, Mohéli, Comoros

**Keywords:** Flying foxes, Monitoring, Seasonality, Population status, Deforestation

## Abstract

Flying foxes of the genus *Pteropus*, especially those inhabiting islands, face increasing pressure from anthropogenic threats. A first step to implementing effective conservation actions is to establish monitoring projects to understand a species’ population status and trend. *Pteropus* species are highly affected by seasonality which further requires regular, repeated, and long-term data to understand population trends, and reactions to severe weather events. In the present case study, a regular, bi-annual population census was implemented on Comoros between 2016 and 2023 for the highly threatened Livingstone’s fruit bat, *Pteropus livingstonii*, and compared the results of standardized monitoring to historical population data. Seasonality had a large impact on the number of bats found at roost sites, with more bats present in the wet season, but the data over the past eight years revealed no significant in- or decrease in the number of bats counted on the island Anjouan. We estimated around 1,200–1,500 bats on Anjouan and 300–400 bats on Mohéli, and found that landcover type has no measurable effect on population distribution at roost sites. Our study highlights the need for long-term surveys to understand past population trends and that single counts are not sufficient to draw final conclusions of a species’ status.

**Supplementary Information:**

The online version contains supplementary material available at 10.1186/s12862-024-02255-w.

## Background

Flying foxes (genus *Pteropus*) are considered an “island taxon” as the majority of the 64 extant species occur on islands of the Indian and Pacific Ocean [1]. Island habitats are particularly vulnerable to destruction and irreversible alterations [2], heavily impacting the entire genus [3, 4]. There is an urgent need to understand population dynamics and trends of flying fox species as their remaining habitat is systematically destroyed across the globe [5–8]. In addition, the effects of urbanisation [9], climate change and increasing tropical storms [10], as well as unsustainable hunting [11, 12], are affecting *Pteropus* populations. While all these factors can potentially lead to a drastic decrease in population sizes and local extinctions, most flying fox species are not monitored at all, surveyed only occasionally, or monitoring has started only very recently, when populations may have already responded negatively, leading to shifted baselines (accepting an already impacted situation as “normal” or the “status quo” [13]).

While various active and passive monitoring methods exist [14], i.e., via camera traps, acoustic recorders, mark-recapture [15], tagging [16], direct point counts [17], transects [18], eDNA samples [19] or citizen science [20], there are considerable difficulties to consider when it comes to monitoring flying foxes as the animals are mobile, nocturnal, often roost in inaccessible areas, or the bats may be easily disturbed when approached [21]. Additionally, flying foxes show a pronounced response to seasonality across their range: *Pteropus voeltzkowi* density is lower during the rainy season [22] and both *P. scapulatus* and *P. alecto* move to satellite camps during periods of fruit abundance in the monsoon season [23, 24]. Similarly, *P. niger* and several other species show differences in travelling distance [25, 26] and population density of species visiting urban areas, such as *P. dasymallus*, also fluctuate across seasons [27]. These fluctuations are a reaction to variations in resource availability although the type of resources have often not been identified, making it difficult to predict population patterns in different context. As such, irregular or single population counts do not provide a full picture of the state of any given *Pteropus* species. Singular counts also give no insight into a population’s reaction to habitat alterations or severe weather events: long-term, standardized data is necessary to draw conclusions of a species population baseline, seasonal effects and conservation concerns [28].

In the present study we wanted to understand how long-term data from regular monitoring can be used to draw conclusions about the population status of the Livingstone’s fruit bat, *P. livingstonii*, on Comoros. This bat species is highly threatened with an estimated global population of around 1,300 individuals occurring on two islands in the Indian Ocean: Anjouan and Mohéli [29], with the larger part of the bat population located on Anjouan, the island with the highest human population density and deforestation rates [30]. The Comoros are some of the most densely populated islands in the world with high rates of deforestation threatening local biodiversity [31]. The natural vegetation on the islands is rainforest, with high levels of endemicity in both fauna and flora, which has prompted the declaration of zones as Important Bird Areas on both islands by Birdlife International [32]. Anthropogenic pressure on forests, exacerbated by poverty, and lack of alternatives to subsistence farming, has led to widespread deforestation, especially on Anjouan where 80% of the natural forests were lost in two decades [30]. To counteract this ongoing forest loss, National Parks have been established on Anjouan and Mohéli in 2018 and 2001 respectively [33]. As the bats are not hunted and have no natural predators on the islands, the main threat they are facing is the continuous deforestation: under pressure from habitat loss, the Livingstone’s fruit bat faces extinction, which could further exacerbate the degradation of the local ecosystem as, being the largest native mammal on the islands, they likely act as a keystone species [1, 34]. Given this pressing situation it is important to understand the current status of the species’ population and how they respond to the ongoing landscape degradation.

We present an eight-year long, targeted monitoring programme by the local NGO Dahari. Dahari was established in 2013 with a mission to support rural communities to restore the ecosystems of the Comoros. Before Dahari, occasional monitoring of the population was done in the early 1990s, and a regular monitoring programme involving local monitors was established by the organisation Action Comores in 1997 and continued until 2006 [35]. The population surveys were re-established in 2011 under the guidance of the NGO Dahari. While data for this species therefore exists since the 1990s, it is difficult to draw comparisons and understand population trends for this period as monitoring was not done regularly, the bats exhibit high seasonality in their presence at roost sites, and more roost sites were added to the surveys over time. Presently, after nearly a decade of regular, bi-annual monitoring with standardized methods, we aim to answer the following questions with this study: (1) can eight years of roost survey data help to detect a past population trend over time despite seasonal differences, and increasing survey location numbers? And (2) does landcover type around the roost play a role in the bats’ distribution across seasons? We aimed to understand whether there are seasonal preferences for certain landcover types, such as forests [36]. We also compare our findings to previously published population numbers to understand how results from regular monitoring can provide information that may be lacking in sporadic, incomplete counts.

## Methods

### Study site and roosts

**Fig. 1:**
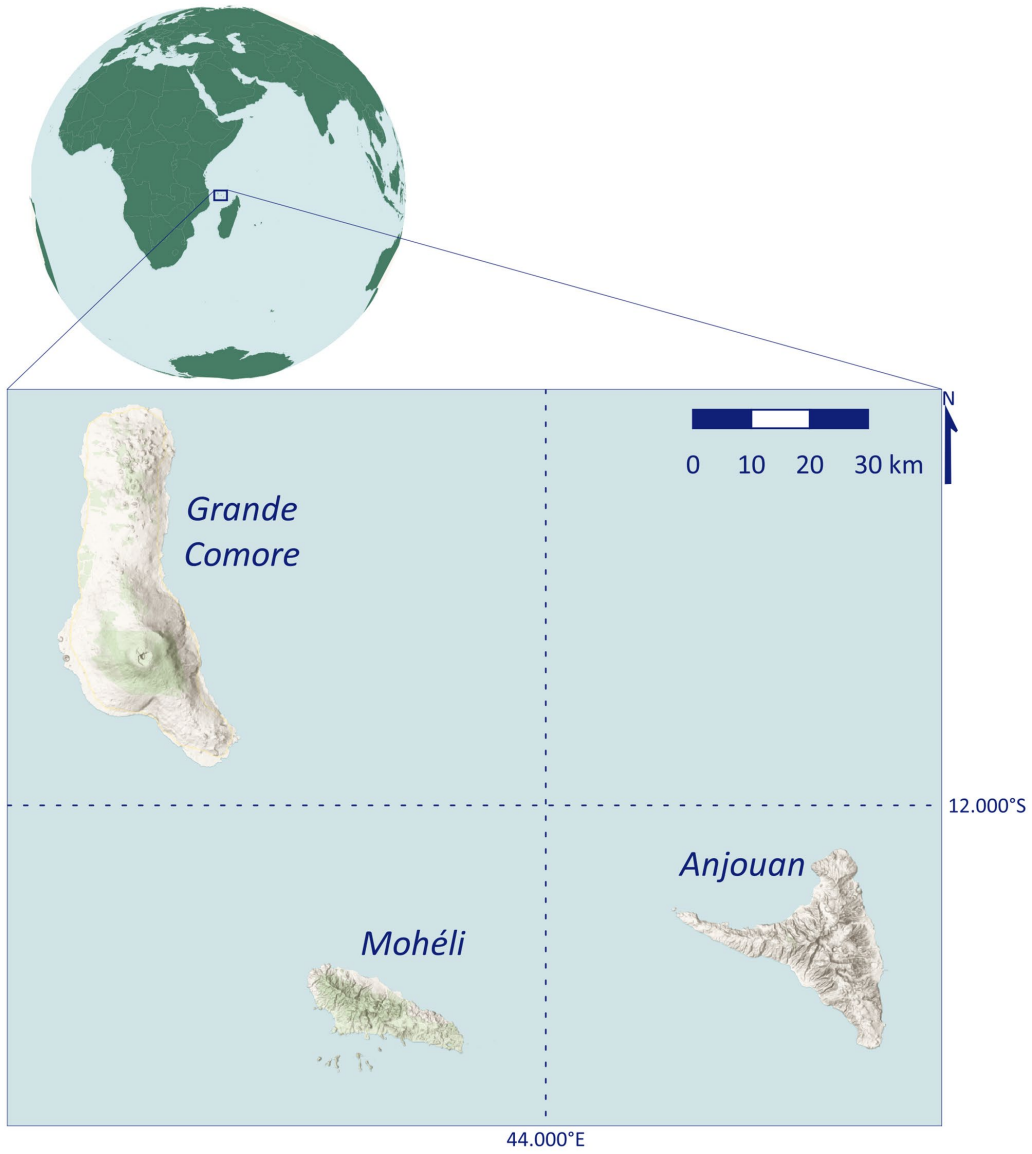
Comoros archipelago, located in the Mozambique Channel. The study was implemented on Anjouan and Mohéli where the Livingstone’s fruit bat, *Pteropus livingstonii*, is endemic

Data were collected between 2014 and 2023 in the natural habitat of *Pteropus livingstonii*: the islands Anjouan (Comorian: Nzwani, S12°14’6”, E044°27’21”) and Mohéli (Comorian: Mwali, S12°18’56”, E043°43’3”), of the Comoros archipelago, located in the Mozambique Channel (Fig. 1). Both islands are small (Anjouan: 424 km^2^, Moheli: 211 km^2^), and mountainous, with Anjouan having a higher elevation profile (highest peak: Mt. Ntringui with 1595 m) than Mohéli (highest peak Mt. Mlédjélé with 790 m). The seasonality in Comoros is marked by a hotter, humid season between November and April (mean daily temperature: 28.2 °C), and a cooler, drier season between May and October (mean daily temperature: 26.0 °C). All 19 known *P. livingstonii* roost sites (status: 2023) are located above 400 m elevation on Anjouan, with most roost sites between 600 and 1000 m. Only eight roost sites (five of which are currently used by *Pteropus livingstonii*) are known from Mohéli, all above 230 m elevation. The 22 currently occupied roosts are located in wooded areas: either natural forests that are degraded to varying degrees or within agroforests (which are marked by a mix of native and non-native fruit and spice trees and crop plants [37]). A single roost may consist of as many as 20 individual trees, and roost trees are generally large, old-growth, native trees but bats will use introduced, non-native, trees as roosts as well [38, 39].

### Population monitoring

While regular population surveys on Mohéli started only in 2019, first population surveys conducted by Dahari were implemented in 2014 and 2015 on Anjouan: in those years the bats were counted over a timespan of a few weeks, which likely led to overestimation of the population due to the high mobility of the bats. The methodology was therefore revised in 2016, and regular, bi-annual (once in the dry season, once in the wet season), population monitoring was implemented. During each survey, all known roosts were visited within two days, counting simultaneously at as many sites as possible to avoid double counting. All bats present at the roost trees were counted by observers using binoculars from vantage points which were located at a mean distance of 70 m from the roost site but could be as close as 30 m or as far as 300 m, depending on the location and accessibility of the site. A minimum of two observers were stationed at each roost site, counting all bats that were present and stationary in the trees, with numbers being recorded on printed datasheets or digitally with handheld tablets. All present bats were counted, as it was difficult to distinguish between mature and immature individuals at a distance once the pups became independent. The bats were counted twice: at 8 AM and 11 AM with all movement between trees, and to and from the roost site recorded in the meantime. Two counts were completed at the designated times as the weather in the mountains is changeable and clouds or fog may cause low visibility for parts of the morning. We chose the morning as bats were observed to start leaving their roosts in the early afternoon. Due to their large size, and the lack of dense vegetation in the roost trees, it is unlikely that many individuals were missed during these counts. For each roost the distance to the nearest agricultural field, the distance to the nearest human-made path, and the surrounding landcover type (natural or degraded forest, agroforest) were recorded and basic weather data (% cloud coverage, % fog cover) estimated. Due to limited equipment, more accurate measurements of environmental variables could not be made. All roost trees that were accessible were marked with GPS points and identified to species level. Due to logistical problems, wet season counts were sometimes conducted early in the following year (January/February, rather than December). If weather conditions were unfavourable (fog, rainfall) and did not allow for precise counting at multiple roosts, the entire count was repeated two weeks later to ensure data was available for the season. We therefore have multiple datapoints for each roost during each seasonal survey (counted multiple times per day, as well as multiple times per season if necessary). Here we report the maximum number of bats for each roost site during the respective surveys. It is possible that these numbers are slightly overestimated due to movements between roosts during the surveys, but we are confident that they are close to the true population size as the survey duration was short (two days). A roost would count as completely abandoned when there were no bats recorded at the site for two years or four consecutive counts. New roost sites were discovered and added to the monitoring from 2019 onwards. In November 2019, the first comprehensive, simultaneous, population survey across both islands (Anjouan and Mohéli) was conducted using the methods described above. The simultaneous counts were then continued from February 2023 onwards. Prior to the count, we identified five roost sites that are currently in use on Mohéli. While the results of 2014 and 2015 are presented in the following sections, we limited the analysis to data collected on Anjouan from 2016 as the revised methods make those counts more robust. We also compared our results to previously published population estimates from surveys conducted between 1992 and 2015.

### Analysis

To understand whether there is a detectable population trend over the past eight years of monitoring despite seasonal differences, and increasing survey location numbers, we used a negative binomial Generalized Linear Mixed Effects Model (GLMM) and evaluated the contribution of fixed effects using a Wald test. We set the year as a fixed effect, with the total number of bats counted in each season (summed across all sites) as the response variable, while controlling for season and number of surveyed locations by setting those as random effects. To answer our second question, if bats prefer to roost in specific landcover types during either the wet or the dry season, we also used a negative binomial GLMM to investigate the relationship between the number of bats present at roost sites located either in natural forest, degraded forest, or agroforest across both seasons: for this we set the landcover type and season, as well as the interaction between these factors, as fixed effects, while controlling for variation over time and between indidivual roost sites by setting roosts and year as random effects. Pairwise comparisons were computed using Tukey HSD tests to identify significant differences between variables. As the conditional variance exceeded the conditional mean of the data, negative binomial GLMMs were deemed the appropriate approach for analysis and model fit was assessed with a diagnostic report. We performed all analysis using the statistical software R [40], fitting the models with the packages ‘lme4’ [41] and ‘report’ [42]. [Model output available as supplementary material.]

## Results

### Population monitoring and seasonality

**Fig. 2:**
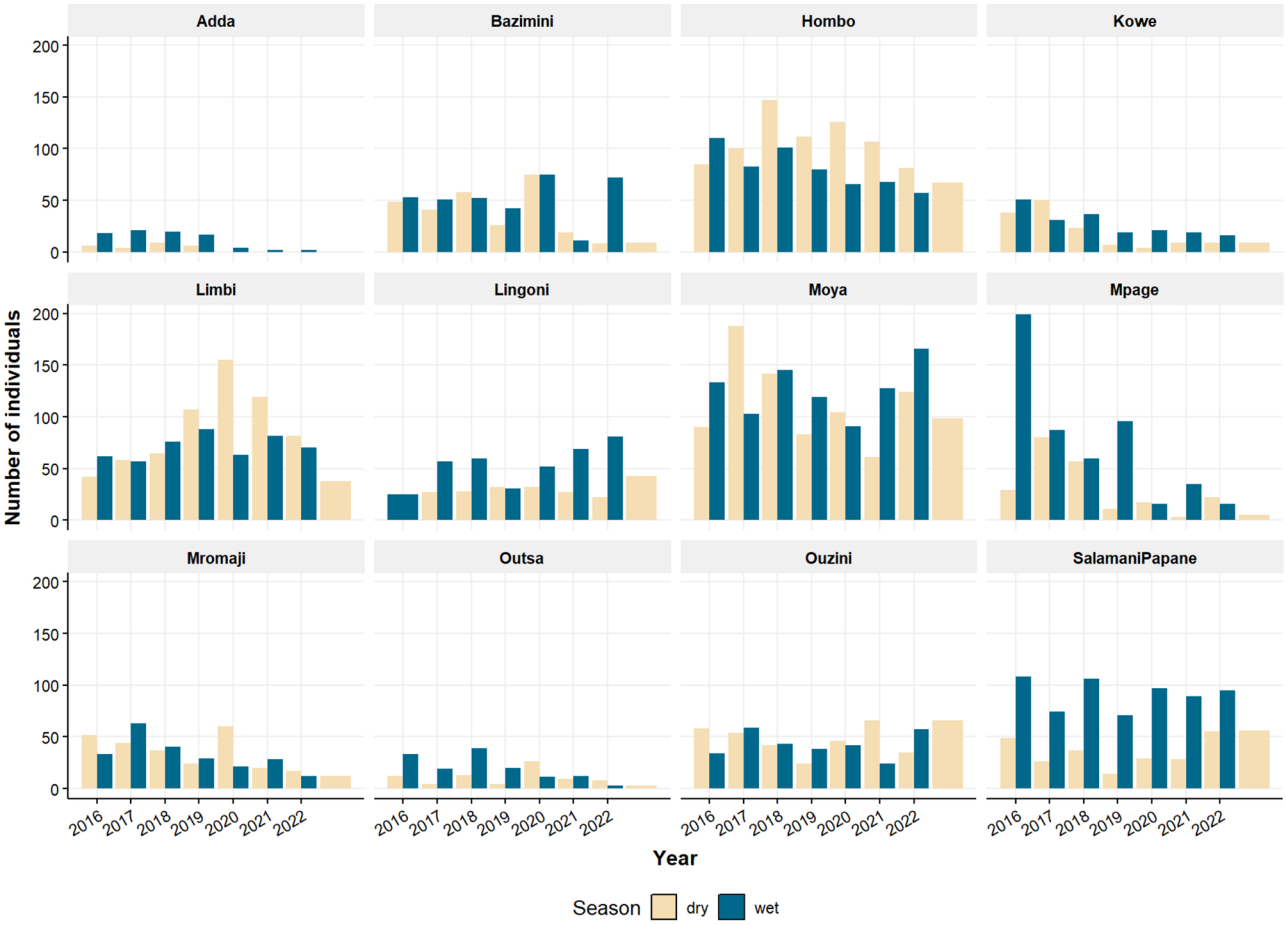
Seasonal fluctuations in Livingstone’s fruit bat colony sizes at 12 long-term roost sites on Anjouan, Comoros, between 2016 and 2022

The bi-annual population surveys on Anjouan revealed consistent high seasonality in the numbers of *P. livingstonii* individuals at roost sites: the number of individuals found across roost sites was higher during the wet season (mean ± SD: 949.1 ± 270.4) than during the dry season (635.7 ± 81.1). The mean (± SD) number of bats found at roosts during the dry season was 40.3 (± 41.3), while the colony size increased to a mean of 59.5 (± 96.5) individuals during the wet season. Individual roost sites, however, showed contrasting patterns: whereas Moya (agroforest) and Ouzini (degraded forest) harbour a similar number of bats all year round, in Hombo (natural forest), Limbi (natural forest) and Kangani Bwejou (agroforest) we recorded more bats during the dry than the wet seasons (Fig. 2). Over the years, survey locations increased from 14 to 17 on Anjouan as more roost were discovered, as well as some abandoned (Table 1). Tropical cyclones (usually occurring between January and April) had large impacts on the individuals present at roost sites as the population dropped to an all-time low (481 individuals) directly after cyclone “Kenneth” hit the island in 2019, but the numbers slowly recovered over the next 12 months. From 2020 onwards a discovery of a large new roost led to a rapid increase in total population size, more than doubling the previous records during the wet season counts to a total of 1489 individuals on Anjouan alone. Data for the Mohéli population is limited to surveys in November 2019 with 170 individuals, the 2022 wet season count where we recorded 228 individuals, and the 2023 dry season count with 345 individuals (Table 2).

**Fig. 3:**
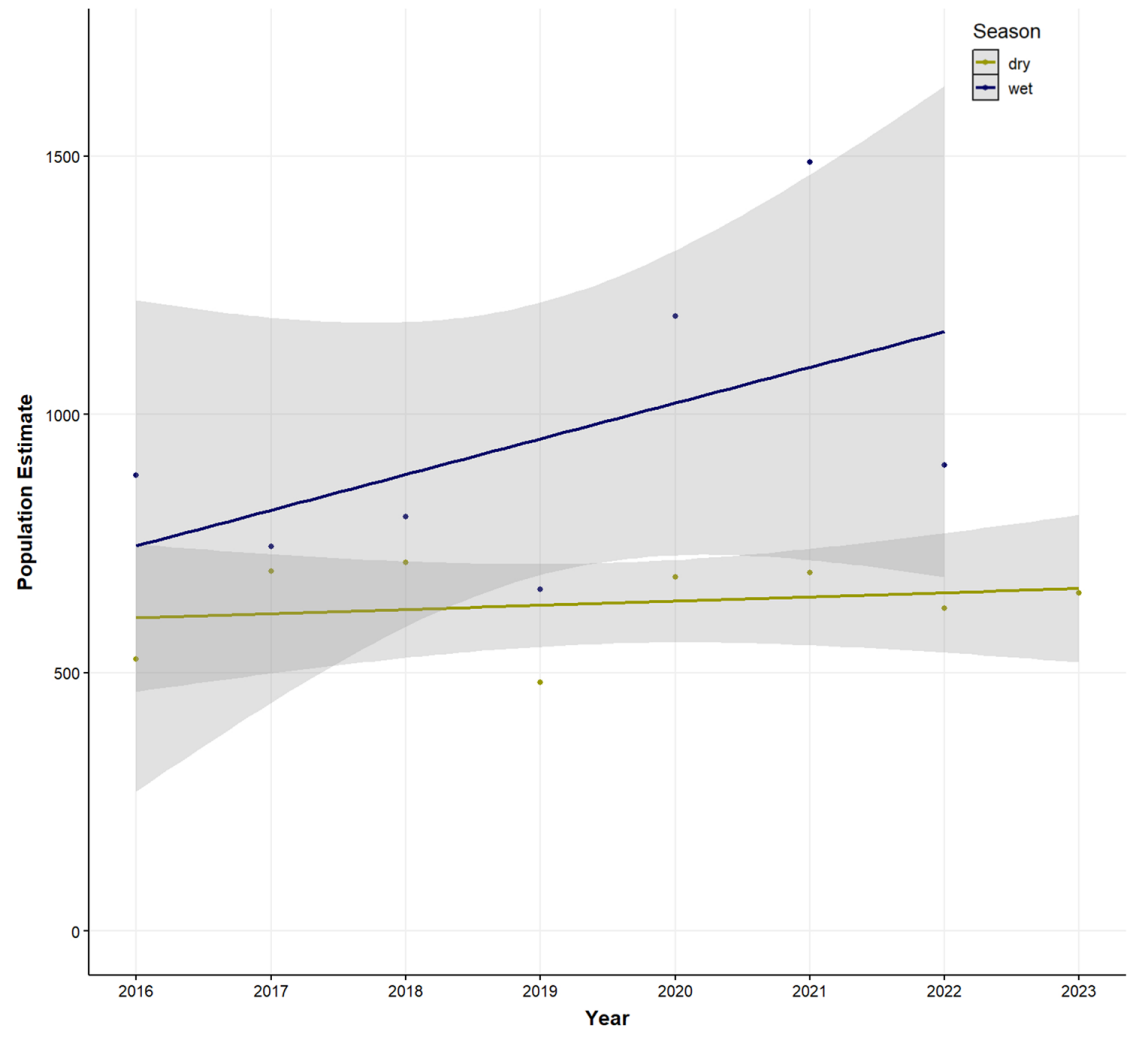
Number of *Pteropus livingstonii* individuals counted bi-annually at roost sites between 2016 and 2023 on the island Anjouan, Comoros. Different colours represent different seasons: dry season = May to October, wet season = November – April. Grey shading illustrates confidence interval. Regression lines are not significant

The most recent population surveys for Anjouan and Mohéli combined resulted in 1130 individuals during the 2022 wet season and 999 individuals during the 2023 dry season. For the Anjouan population, there was no statistically significant effect of year on the reported population (Anova: X^2^_(1)_ = 0.27, *p* = 0.59) (Fig. 3), highlighting that the population has been stable over the past eight years.

**Table 1:** Maximum observed number of Livingstone fruit bat individuals for each roost site on Anjouan, Comoros, during each survey since 2016. Landcover type has been classified as AG = agroforest, FD = degraded forest or FN = natural forest. Mean number of trees represents the average number of trees used by the bats at each site between 2016 and 2023. Missing values indicate no survey took place

Roost (elevation in m)	Landcover type	Mean # of trees	Year Season (Month)														
			2016		2017		2018		2019		2020		2021		2022		2023
			Dry (May/Jun)	Wet (Dec)	Dry (May)	Wet (Dec)	Dry (May)	Wet (Jan 2019)	Dry (Aug)	Wet (Dec)	Dry (Jun)	Wet (Dec)	Dry (Jul)	Wet (Dec)	Dry (Jul)	Wet (Feb 2023)	Dry (Jul)
Adda *(980 m)*	AG	*3.4*	6	18	4	21	9	20	6	17	0	4	0	2	0	2	0
Bazimini *(752.7 m)*	FD	*10.7*	49	53	41	51	58	52	26	42	75	75	19	11	8	72	9
Dindri *(777.2 m)*	AG	*3.2*	0	4	4	10	2	4	0	0	0	0	0				
Hombo *(786.3 m)*	FN	*11.7*	85	110	100	83	147	101	112	80	126	66	107	68	81	57	67
Kangani Bwejou *(630.4 m)*	AG	*4.6*	16	15	16	20	53	0	31	11	12	3	26	17	9	3	15
Kangani Kove *(557.3 m)*	FD	*1*	1	4	0	0	0	19	0	0	0	0					
Kowe *(584.9 m)*	AG	*8*	38	51	50	31	23	37	7	19	4	21	9	19	9	16	9
Limbi *(960.5 m)*	FN	*4.5*	42	62	58	57	65	76	107	88	155	63	119	82	82	70	38
Lingoni *(726.7 m)*	FN	*4.9*		25	27	57	28	60	32	31	32	52	27	69	22	81	43
Moya *(622.5 m)*	AG	*12.5*	90	133	188	103	142	145	83	119	104	91	61	128	124	166	99
Mpage *(739.9 m)*	FN	*5.1*	29	199	80	87	57	60	11	96	17	16	3	35	22	16	5
Mromaji *(434 m)*	AG	*6.2*	52	33	44	63	37	40	24	29	60	21	20	28	17	12	12
Outsa *(601.2 m)*	AG	*2*	12	33	4	19	13	39	4	20	26	11	9	12	8	3	3
Ouzini *(920.4 m)*	FD	*11.7*	58	34	54	59	42	43	24	38	46	42	66	24	35	57	66
Salamani Papane *(791 m)*	AG	*9.25*	49	108	26	74	37	106	14	71	29	97	28	89	55	95	56
Adda-Ouzini *(1004.1 m)*	FD	*4.5*											7	0	12	4	4
Adda (Hampouhou) *(677.8 m)*	AG	*3.5*											18	12	17	23	31
Kowe 2 *(1069 m)*	FN	*2.3*											126	52	31	80	19
Mpage (Moija) *(402.5 m)*	AG	*16.6*										628	49	841	93	145	178
		**Total**	**527**	**882**	**696**	**735**	**713**	**802**	**481**	**661**	**686**	**1190**	**694**	**1489**	**625**	**902**	**654**

**Table 2:** Maximum observed number of Livingstone fruit bats for each roost site on Mohéli, Comoros, since 2019. Landcover type has been classified as AG = agroforest, CR = crop field, FD = degraded forest or FN = natural forest. Mean number of trees represents the average number of trees used by the bats at each site between 2019 and 2023. Missing values indicate no survey took place

Roost (elevation in m)	Landcover type	Mean # of trees	Year Season (Month)		
			2019	2022	2023
			Wet (Nov)	Wet (Feb 2023)	Dry (Jul)
Barakani (*522 m*)	FN	*3*		50	84
Hanakulemba (*479 m*)	FD	*10*	62	52	138
Hasserandrengué (*381 m*)	FN	*3*	39	66	80
Kidogobasse (*231 m*)	AG	*4*	21	60	21
Mlédjélé (*480 m*)	AG	*5*	48		
Tredjani *(227 m)*	CR	*2*			22
		Total	170	228	345

**Fig. 4:**
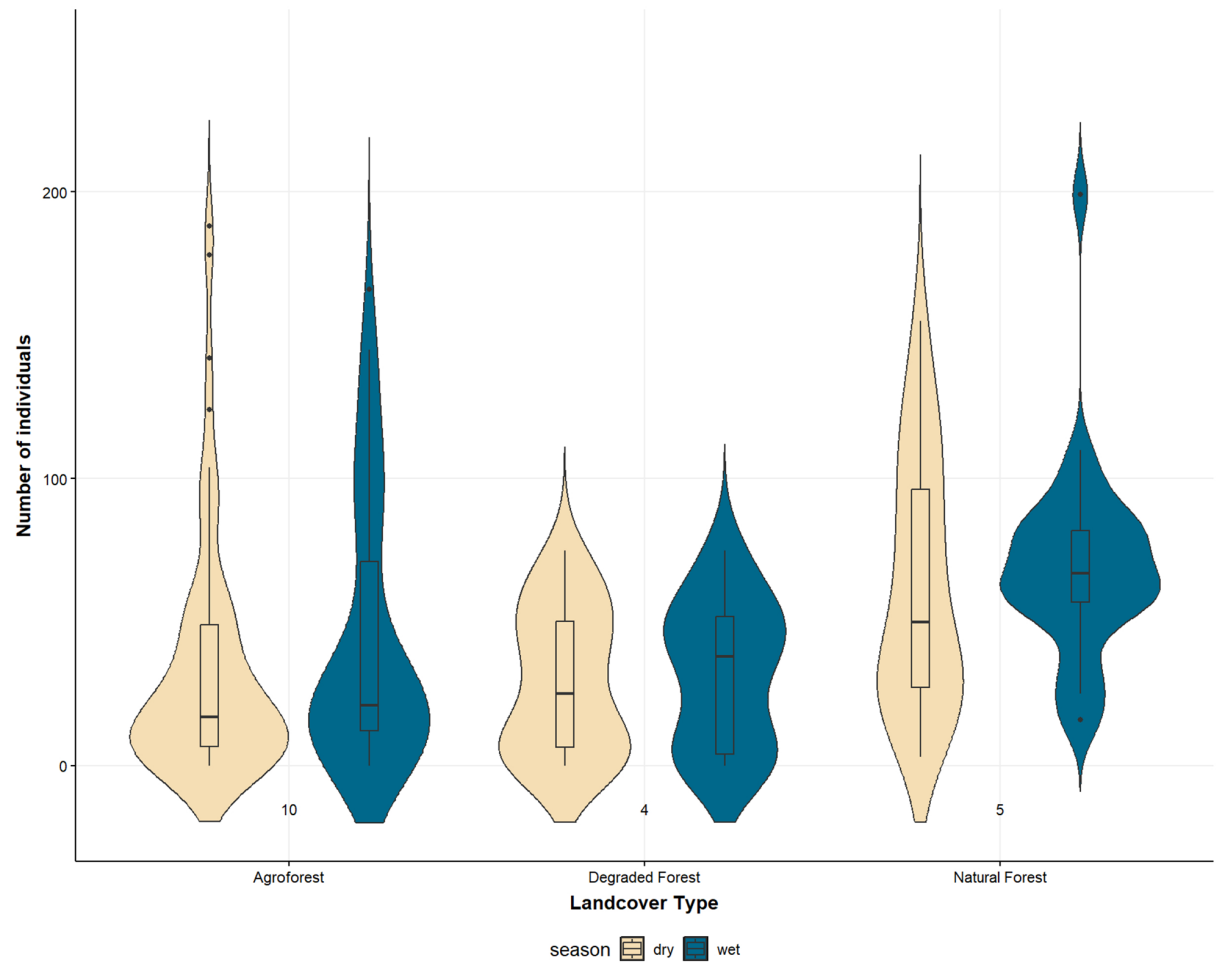
Number of Livingstone’s fruit bats in roosts located in different landcover classes on the island Anjouan, Comoros, between 2016 and 2023. Boxplots represent the distribution of the first and third quartile around the median. Sample size = number of roosts, (N), is noted at bottom of plots

We did not find seasonal preferences for a specific landcover type in the data: while season had a significant effect (Anova: X^2^_(1)_ = 13.81, *p* < 0.001) in our model investigating the interaction between landcover type and season, with more bats being present during the wet season, there was no overall statistically significant effect of landcover type on the bats’ distribution (Anova: X^2^_(2)_ = 4.12, *p* = 0.12), nor on the interaction between landcover type and season (Anova: X^2^_(2)_ = 1.32, *p* = 0.51) (Fig. 4). Pairwise comparisons also showed no significant differences between landcover types across seasons.

### Comparison to historical data

**Fig. 5:**
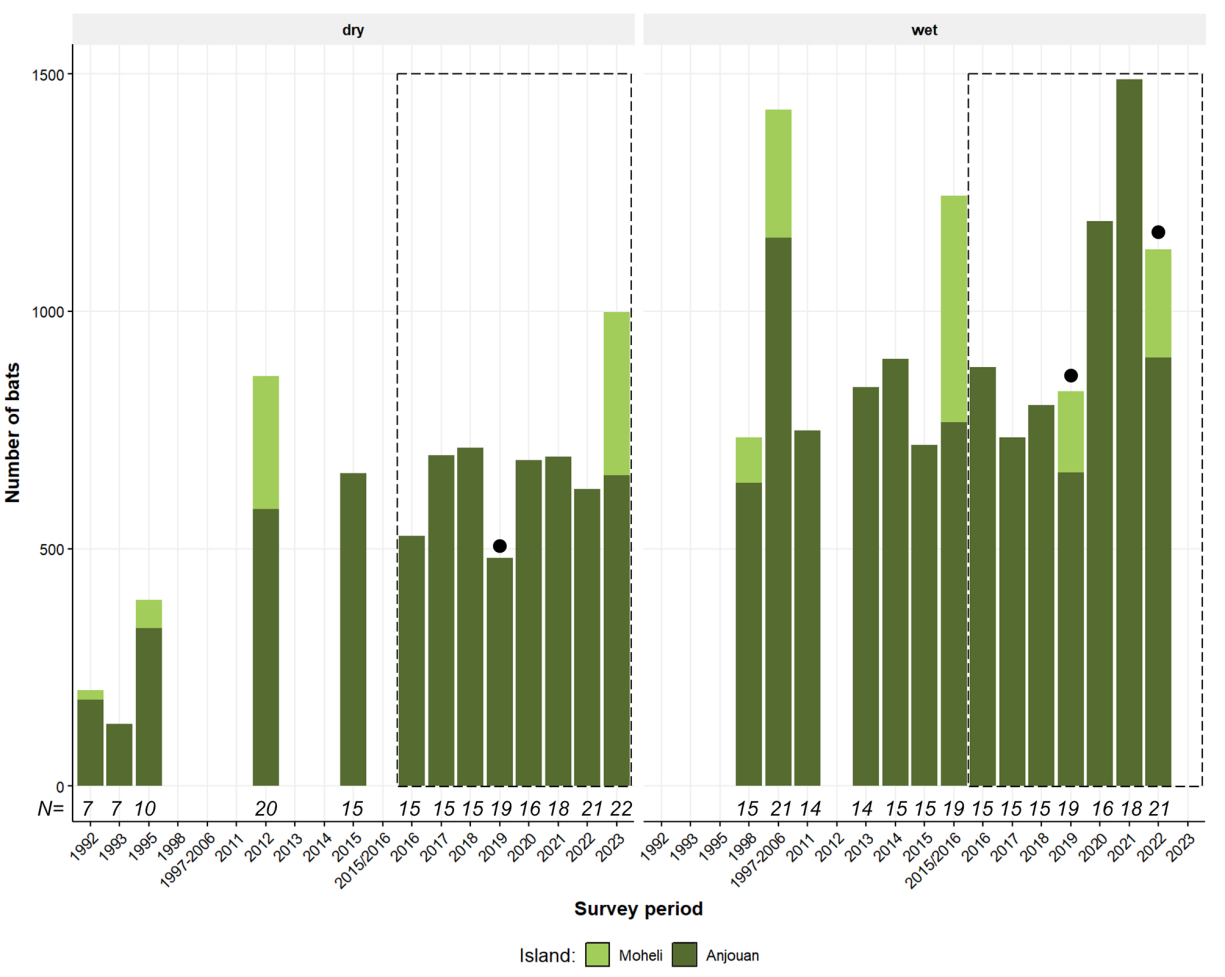
Total number of Livingstone’s fruit bats recorded in surveys on the Comoros since 1992, split by season. Dry season = May – October, wet season = November - April. The data between the time points 1992 and 2015/2016 were extracted from previously published studies (with the exception of 2014 and 2015, which were conducted by the NGO Dahari and not published to date). The data point 1997–2006 represents an average of the reported population for this time period [35] as exact data were not available. The dashed rectangle shows Dahari’s bi-annual standardized monitoring results since 2016. Sample size *N* indicates the number of roost sites surveyed. Dots (•) mark years affected by cyclones since 2016

First surveys of the species were conducted in the early 1990s, when less than 200 individuals were found at seven roosts across both islands [34]. This increased to over 300 individuals at nine roosts for the surveys in 1995 and 1996 [43]. Six more roosts were identified in 1998, increasing the number of bats surveyed to 734 on both islands [39]. The surveys were continued the following years until 2006 by trained local monitors, overseen by the organisation Action Comores and the results were summarised in the Conservation Action Plan for the species [35], the numbers presented below are an estimate based on the average of the reported numbers. The first irregular surveys by Dahari (at 14 roost sites on Anjouan and 5 on Mohéli) were conducted between 2011 and 2013, with population estimates ranging between 650 and 841 individuals, and published in Daniel et al. (2017). Dahari then continued monitoring the species bi-annually on Anjouan, with the first non-standardized surveys in the years 2014 and 2015 recording between 841 (2014 wet season), 900 (2015 wet season) and 659 (2015 dry season) individuals at 15 surveyed roosts. Ibouroi et al. (2018) undertook a comprehensive population count across both islands in 2015 and 2016 and surveyed a total of 19 roost sites, counting 1249 individual bats. While there are seasonal fluctuations visible in the data (Fig. 5), the average number of bats found per roost was 47.6 ± *65.3* (mean ± *StD*) until 2016. During Dahari’s years of regular monitoring the average number of bats per roost increased only slightly to 50.2 ± *76.1* (mean ± *StD*) throughout the years.

## Discussion

Recording data regularly over a longer period not only aids in understanding the effect of environmental factors on population numbers but also allows for identification of population trends [26, 28]. We found that the Livingstone’s fruit bat population showed no significant in- or decrease over the past eight years of monitoring on the island Anjouan where the larger of the two remaining wild populations is located. While the numbers presented here are exact records of the bats found at roost sites during surveys, we cannot exclude the possibility of more bats roosting across the landscape in temporary or undiscovered roosts. Given the difficulty of detecting all bats during any given survey, we currently estimate between 1,200 and 1,500 bats on Anjouan, and 300–400 bats on Mohéli. However, there are significant fluctuations in number of bats present at roost sites across different seasons, with more bats being present during the wet season (November – April). Interestingly, we did not find a preference for roosts located in a specific landcover type (such as natural forest or agroforest), and seasonality remained the only strong predictor for fluctuations in population numbers in our data. In addition, population numbers at roost sites dropped directly after severe weather events such as cyclones (2019 – “Kenneth”, 2022 – “Cheneso”), but recovered in the 12 months following such storms. In the study period, we recorded roost abandonments and identified new roost sites which illustrated the need for regular bi-annual surveys, to understand the bats’ population evolution. Had only one or two irregular surveys been conducted, the seasonality, weather events, and roost site changes would have been missed, likely leading to a skewed picture of the species’ population size. Although such in-depth data from Mohéli are currently not available, due to joint efforts between Dahari and the Mohéli Marine National Park, a long-term bi-annual population survey has been set-up in 2022 on the island, that will allow for a complete picture of the species’ population in their entire native range in the future.

What has been noteworthy is the discovery of new roost sites which, while being used throughout the year, show large fluctuations in colony sizes between seasons. In one the case discovery of a site with over 600 individuals in 2020 led to a drastic increase in the total number of bats found on Anjouan. That this augmentation was brought on by a migration of bats to Anjouan from Mohéli directly after the cyclone in 2019 is unlikely: while the species could cross the ∼ 60 km distance between the islands [36, 44], genetic studies have shown there to be little exchange between the two populations [45, 46]. It is more likely that the increased population recoded on Anjouan in the past three years is due to an increased number of roost sites being identified through GPS-tracking of bats [47] and communication with landowners who found bats on their land.

The roosting habits of the bats (singly or in small groups) and the mountainous terrain of the islands have made detecting new sites difficult, especially in times of resource scarcity the bats disperse in the landscape, roosting singly near feeding sites, often in difficult to access areas. On Comoros the hypothesized time of food scarcity is the dry season, which coincided with fewer bats present at roost sites. The seasonal fluctuations in flying fox distributions have been attributed to variation in feeding resources within a landscape [22, 25, 48, 49]. While the feeding resources for this species are not fully identified, prior research points towards a dependence on native forest tree species [35, 50]. As these trees are found mainly in natural and degraded forest habitat [37], we used these landcover types as a proxy for resource availability and investigated whether bats moved to roosts located in natural forests during the dry season. As we found no effect of landcover type on roosting behaviour we suggest that there is a possibility of feeding trees being distributed throughout the island, regardless of landcover type. Elevation as a driving factor for roost selection has been suggested for the Livingstone’s fruit bats [38, 51] but it wasn’t possible to dissect the effects of landcover type and elevation as the remaining forests are all located in higher elevations.

Comparison to historical data has proven more complex as the very low numbers reported in the first surveys can either stem from fewer roosts being surveyed, the surveys being conducted in the dry seasons, or the population having grown naturally since then. These factors are not mutually exclusive but difficult to assess. Similar to other *Pteropus* species, Livingstone’s fruit bats give birth to a single pup per year, reach sexual maturity between 2 and 3 years of age, and not all adult individuals are sexually active (G. Glendewar, person. comm.). Considering the limited resources available in degraded habitats, together with high mortality rates, wild flying fox populations generally have a very low natural capacity to increase [52], and it is therefore implausible that the substantial population increase from around 200 individuals in the early 1990s to more than 1500 bats thirty years later, amidst ongoing habitat destruction and no effective protection measures, is due to natural population growth alone. The present study found no significant population increase over time, indicating that the population has been stable this past decade.

The NGO Dahari bases conservation decision-making on the results of these surveys, namely which roost sites to prioritise for protection, and the long-term nature of the collected data allows for better understanding of population trends and the effectiveness of such protective measures [28]. From a conservation perspective, long-term monitoring data can give an excellent indication of a species’ baseline, despite natural fluctuations [28], and enables practitioners and scientists alike to react to any noticeable decreases, increases, or conflicts with the human population in a timely way [53, 54]. For flying foxes in particular the methods used can be easily applied in different contexts, as low-tech equipment such as binoculars, and vantage points that minimise the disturbance to the bats during the day are likely to be found across most flying fox ranges. The methods also require no initial skills and minimal literacy but can always be expanded to include more complex measurements or the use of survey application on mobile devices, allowing for adaptation to local circumstances and training in scientific skills [55]. Depending on the context, the set-up and maintenance of the monitoring scheme can be cost- and labour-intensive, especially when roost sites have to be identified beforehand, which requires additional survey effort. As such, long-term monitoring projects are likely to be most successful when implemented by associations or organisations that are able to source funding and know-how and provide a secure basis for monitors to operate out of [56]. The benefits of regular population surveys in the long-term, given that the collected data answer the initial questions [28], greatly outweigh the costs, especially for flying foxes where monitoring is urgently needed [57, 58]. Regular population surveys are the basis to understanding the evolution of flying fox populations, their responses to the increasing habitat loss and human encroachment. Especially on islands, where there is little to no alternative habitat for the bats to move to [8], regular monitoring allows us to identify problems as well as opportunities for the conservation of this highly threatened taxa.

## Conclusions

The results of this study highlight the need of long-term monitoring for highly mobile animals whose distribution patterns are affected by seasonality. Single, irregular population surveys would not paint a complete picture of the status of the population, and seasonal increases at surveys sites could be misinterpreted. Similarly, the effects of severe weather events, or other environmental factors, could be over- or underestimated if population monitoring was not performed regularly. Regular surveys also allow conservation organisations and practitioners to act in a timely and informed manner to observed changes in the estimated population. With relatively low-tech methods, the presented monitoring study can easily be applied to other flying fox populations across their range, as high anthropogenic pressure threatens multiple species across the globe.

### Electronic supplementary material

Below is the link to the electronic supplementary material.

Supplementary Material 1

## Data Availability

The datasets generated and/or analysed during the current study are available from the NGO Dahari on reasonable request.
