# Supplementary material for: Seasonal trends and population status of the highly threatened Pteropus livingstonii in the Comoros archipelago
Source: BMC Ecol Evol. 2024 May 19;24:64. doi: 10.1186/s12862-024-02255-w (PMC11103843; doi:10.1186/s12862-024-02255-w)
Supplement: Supplementary file 1 — Supplementary Material 1 [file 12862_2024_2255_MOESM1_ESM.docx]

Supplementary Materials I

**Title:** Seasonal trends and population movement of the highly threatened *Pteropus livingstonii* in the Comoros archipelago

**Author information**

Isabella Mandl*, Amelaid Houmadi, Ishaka Said, Badrane Ben Ali Abdou, Nastazia Mohamed, Abdoulkader Fardane, Samirou Soulaïmana, Misbahou Mohamed, Ben Anthoy Moussa, Hugh Doulton

*[isabella.mandl@univie.ac.at](mailto:isabella.mandl@univie.ac.at)

Tab.I.1. Model parameters and results for the two negative binomial Generalized Linear Mixed Models used to analyse effects of season, year, and landscape cover on the Livingstone’s fruit bat population of Anjouan, Comoros.

| **Model 1:** count~year + (1 \| season) + (1 \| number of surveyed roosts) | | | | | | |  |
| --- | --- | --- | --- | --- | --- | --- | --- |
| Control: glmerControl(optimizer = "bobyqa") | | | | | | |  |
| AIC: 204.4, R^2^ = 0.89 |  |  |  | |  | |  |
| ***Fixed Effects:*** | ***Estimate*** | ***Std. Error*** | | ***z value*** | | ***Pr(>\|z\|)*** | |
| (Intercept) | 6.84 | 0.26 | 26.05 | | <2e-16 *** | |  |
| year | -0.01 | 0.03 | -0.52 | | 0.59 | |  |
|  |  |  |  | |  | |  |
| **Model 2:** count~landscape*season+(1 \| roost) + (1 \| year) | | | | | | |  |
| Control: glmerControl(optimizer = "bobyqa") | | | | | | |  |
| AIC: 2086.3, R^2^ = 0.84 |  |  |  | |  | |  |
| ***Fixed Effects:*** | ***Estimate*** | ***Std. Error*** | ***z value*** | | ***Pr(>\|z\|)*** | |  |
| (Intercept) | 2.98 | 0.39 | 7.65 | | 1.97e-1 *** | |  |
| Natural forest | 1.03 | 0.65 | 1.57 | | 0.11 | |  |
| Degraded forest | -0.55 | 0.71 | -0.77 | | 0.44 | |  |
| Wet season | 0.42 | 0.12 | 3.50 | | 0.00* | |  |
| Natural forest : wet season | -0.19 | 0.19 | -0.99 | | 0.32 | |  |
| Degraded forest : wet season | -0.20 | 0.24 | -0.86 | | 0.38 | |  |

**Results of the pairwise comparisons of the factors in Model 2**

Results are given on the log (not the response) scale. Confidence level used: 0.95

P value adjustment: Tukey method for comparing a family of 6 estimates

|  | ***estimate*** | ***SE*** | ***df*** | ***z.ratio*** | ***p.value*** |
| --- | --- | --- | --- | --- | --- |
| Agroforest (dry)-Degraded Forest (dry) | 0.55 | 0.71 | *Inf* | 0.77 | 0.97 |
| Agroforest (dry) - Natural Forest (dry) | -1.03 | 0.65 | *Inf* | -1.57 | 0.61 |
| Agroforest (dry) – Agroforest (wet) | -0.42 | 0.12 | *Inf* | -3.50 | 0.00* |
| Agroforest (dry) -Degraded Forest (wet) | 0.33 | 0.71 | *Inf* | 0.46 | 0.99 |
| Agroforest (dry) – Natural Forest (wet) | -1.26 | 0.6 | *Inf* | -1.92 | 0.38 |
| Degraded Forest (dry) – Natural Forest (dry) | -1.59 | 0.80 | *Inf* | -1.96 | 0.36 |
| Degraded Forest (dry) – Agroforest (wet) | -0.98 | 0.717 | *Inf* | -1.36 | 0.74 |
| Degraded Forest (dry) – Degraded Forest (wet) | -0.22 | 0.207 | *Inf* | -1.07 | 0.89 |
| Degraded Forest (dry) – Natural Forest (wet) | -1.82 | 0.811 | *Inf* | -2.24 | 0.21 |
| Natural Forest (dry) – Agroforest (wet) | 0.61 | 0.658 | *Inf* | 0.93 | 0.93 |
| Natural Forest (dry) – Degraded Forest (wet) | 1.37 | 0.812 | *Inf* | 1.68 | 0.53 |
| Natural Forest (dry) – Natural Forest (wet) | -0.22 | 0.16 | *Inf* | -1.43 | 0.70 |
| Agroforest (wet) – Degraded Forest (wet) | 0.75 | 0.719 | *Inf* | 1.05 | 0.89 |
| Agroforest (wet) – Natural Forest (wet) | -0.84 | 0.659 | *Inf* | -1.27 | 0.79 |
| Degraded Forest (wet) – Natural Forest (wet) | -1.60 | 0.813 | *Inf* | -1.96 | 0.36 |
